# Nest predation of Cory's shearwater *Calonectrisborealis* (Aves, Procellariiformes) by introduced mammals on Terceira Island, Azores

**DOI:** 10.3897/BDJ.11.e112871

**Published:** 2023-12-22

**Authors:** Lucas Lamelas-Lopez, Marco Ferrante, Paulo A. V. Borges, Isabel Amorim do Rosário, Veronica Neves

**Affiliations:** 1 cE3c- Centre for Ecology, Evolution and Environmental Changes/Azorean Biodiversity Group, CHANGE – Global Change and Sustainability Institute, School of Agricultural and Environmental Sciences, University of the Azores, Rua Capitão João d´Ávila, Pico da Urze, 9700-042, Angra do Heroísmo, Azores, Portugal cE3c- Centre for Ecology, Evolution and Environmental Changes/Azorean Biodiversity Group, CHANGE – Global Change and Sustainability Institute, School of Agricultural and Environmental Sciences, University of the Azores, Rua Capitão João d´Ávila, Pico da Urze, 9700-042 Angra do Heroísmo, Azores Portugal; 2 Functional Agrobiodiversity, Department of Crop Sciences, University of Göttingen, Göttingen, Germany Functional Agrobiodiversity, Department of Crop Sciences, University of Göttingen Göttingen Germany; 3 Ocean Sciences Institute - Okeanos, Department of Science and Technology, Azores University, 9901-862, Horta, Azores, Portugal Ocean Sciences Institute - Okeanos, Department of Science and Technology, Azores University, 9901-862 Horta, Azores Portugal

**Keywords:** biological invasions, camera traps, invasive species, nest characteristics, oceanic island, seabirds

## Abstract

The Azores holds the largest population of Cory's shearwater *Calonectrisborealis* (Cory, 1881) (Aves, Procellariiformes) in the world. Apart from a few mammal-free islets, the bulk of the population breeds in coastal areas on the main human-inhabited islands, where several non-native predators have been introduced. Throughout the entire year of the 2019 breeding season, we used motion-triggered cameras and regularly visited three colonies of Cory's shearwater to identify nest predators and the factors affecting nest predation. A total of 292,624 photos were obtained, of which 97.7% were of Cory’s shearwaters, 1.7% of non-target species (e.g. other birds, rabbits) and 0.52% of potential predators. Of the monitored nests, 25.7% were predated (n = 9), mainly by black rats (n = 8), but also by cats (n = 1). The relative abundance of black rats in the nests was the main factor explaining nest mortality. This variable was significantly and negatively related with the daily survival rate of Cory’s shearwater nestlings. Identification of the main nest predators is crucial for the management and conservation of native bird populations, particularly on oceanic islands, which harbour an important number of threatened and endemic species.

## Introduction

Biological invasions together with habitat degradation and climatic changes are currently the most important biodiversity erosion drivers and a significant component of human-caused global environmental change (Vitousek 1988, Vitousek et al. 1997). Despite an increasing awareness of these threats, namely of biological invasions, the rate of spread of species outside their native range is not slowing down (Seebens et al. 2017). Oceanic island communities are extremely susceptible to invasive species and the dramatic consequences of invasions for native biodiversity are well known (Oppel et al. 2010, Russell et al. 2017). Island communities are characterised by low species richness, but a high rate of endemism and invasive species threaten the existence of species that are not present elsewhere in the world (Williamson 1996, Whittaker and Fernández-Palacios 2007, Russell et al. 2017). Invasive mammals, especially opportunist predators, are the most damaging non-native animals for islands worldwide (Medina et al. 2013, Dawson et al. 2014, Doherty et al. 2016, Russell et al. 2017). Most introduced mammal species were established on oceanic islands during the European colonisation (Vitousek 1988). Invasive mammal predators are implicated in the decline or extinction of more than 700 species of island native vertebrates (Doherty et al. 2016). Particularly, colonial nesting seabirds have been severely affected by the introduction and spread of non-native mammal predators (Bicknell et al. 2009).

The Azores Archipelago, located in the North Atlantic Ocean, is one of the most isolated archipelagos worldwide and a high priority area for seabird conservation. Regular breeding species are the Monteiro's storm-petrel (*Hydrobatesmonteiroi* (Bolton et al., 2018)), an endemic bird considered as Vulnerable (Bolton et al. 2008, BirdLife-International 2018, BirdLife-International 2023), the band-rumped storm-petrel (*Hydrobatescastro* (Harcourt, 1851)), the Bulwer's petrel (*Bulweriabulwerii* (Jardine & Selby, 1828)), the Cory’s shearwater (*Calonectrisborealis* (Cory, 1881)), the Barolo's shearwater (*Puffinusbaroli* (Bonaparte, 1851)), the Manx shearwater (*Puffinuspuffinus* (Brünnich, 1764)), the yellow-legged gull (*Larusmichahellisatlantis* Dwight, 1922), the sooty tern (*Onychoprionfuscatus* (Linnaeus, 1766)), the roseate tern (*Sternadougalliidougallii* Montagu, 1813) and the common tern (*Sternahirundohirundo* Linnaeus, 1758) (Del Nevo et al. 1993, Monteiro et al. 1996a, Monteiro et al. 1996b, Monteiro et al. 1999, Bolton et al. 2008). The impacts of mammal predators on seabird populations in the Azores Archipelago have been reported for several seabird species (Monteiro et al. 1996a, Monteiro et al. 1996b, Amaral et al. 2010, Hervías et al. 2013b, Hervías et al. 2013a, Lamelas-López et al. 2021). Additionally, the introduction of mammals in the Azores has probably caused the disappearance of the Fea's petrel (*Pterodromafeae* (Salvadori, 1899)) and the white-faced storm-petrel (*Pelagodromamarina* (Latham, 1790)) and has led to the population decline of other Procellariiform species (Monteiro et al. 1996a). Currently, introduced mammal species are present in all the main nine islands (De León et al. 2005) and are probably one of the most important threats to Azorean native birds (Monteiro et al. 1996a).

Although only one mammal species was present in the Azores before the Portuguese arrival in the 15^th^ century, the Azores noctule *Nyctalusazoreum* (Thomas, 1901) (Frutuoso 1561), nowadays, both rodents (house mouse *Musmusculus* Linnaeus, 1758, black rat *Rattusrattus* (Linnaeus, 1758) and Norway rat *Rattusnorvegicus* (Berkenhout, 1769)) and carnivores (ferret *Mustelafuro* Linnaeus, 1758, weasel *Mustelanivalis* Linnaeus, 1766, feral cat *Feliscatus* Linnaeus, 1758 and feral dog *Canislupusfamiliaris* Linnaeus, 1758) are present. Each of these exotic species potentially represent a threat to native birds, thus identification of the main nest predators and the factors influencing nest predation is crucial for the conservation of such bird species. Moreover, invasive species can interact with each other (e.g. mesopredator release effect; Courchamp et al. (2001), Russell et al. (2009), Hervías et al. (2013b)) and it is, therefore, important to understand the overall impact of several invasive species and the possible consequences of their eradication, before implementing well intentioned conservation programmes.

In the present study, we monitored nests of Cory’s shearwater on three colonies on Terceira Island (Azores) using motion-triggered cameras. Over the last years, this technology has become an important tool for understanding the impact of invasive mammals on seabird breeding success (Edney and Wood 2020). The Cory’s shearwater is one of the most abundant Procellariiforms in the north-east Atlantic Ocean (Cramp and Simmons 1977, Ramos et al. 2003) and the Azores constitute a pivotal conservation area for this species, given that the Archipelago hosts around 188,000 breeding pairs (BirdLife-International 2004), which represents about 60% of the world population (Monteiro et al. 1996a, Hagemeijer and Blair 1997). In the Azores, Cory’s shearwaters return to the colonies to breed by February-March, laying a single egg in May, chicks hatch by late July-early August and fledge by the second half of October up to early November (Monteiro et al. 1996b). Nest predation is considered the highest cause of land mortality for Cory’s shearwater populations. Previous studies indicate the negative effects of invasive species on Cory’s shearwater survival in the Azores (Hervías et al. 2012, Hervías et al. 2013b, Hervías et al. 2013a). However, they also highlight complex responses where several exotic species interact and have both positive and negative effects on the bird population (Hervías et al. 2013).

Our objectives were: (1) to identify Cory’s shearwater nest predators and (2) to determine the abiotic and biotic factors that may directly or indirectly affect nest predation. In particular, we measured variables related with nest characteristics, as the maximum width and height of the cavity entrance, the nest entrance area, the nest depth and the amount of nest entrance coverage by vegetation; and surrounding habitat, as cover of tree and shrubs, herbaceous plants, rocky areas and barren areas within a 25 m radius of the nest, as well as distance to the nearest urban areas, according to another studies about nest predation in the Azores (Hervías et al. 2013b, Lamelas-López et al. 2020a). Additionally, we estimated the activity of predators and Cory’s shearwaters using Relative Abundance Indices. We expected that predation will be mainly affected by predators’ activity (i.e. relative abundance). In relation to the environmental characteristics, we hypothesised that most accessible nests, i.e. wider nests in uncovered and highly visible areas, will be more predated than narrower nests or with vegetation at their entrance.

## Material and methods

### Study area

The Azores Archipelago is a group of nine volcanic islands and 26 islets, located in the North Atlantic Ocean about 1500 km from Europe and 1900 km from North America (37° to 40° N latitude and 25° to 31° W longitude). The Archipelago is divided into three groups, the Western group, formed by Corvo and Flores Islands, the Central group, formed by Faial, Pico, São Jorge, Graciosa and Terceira Islands and the Eastern group, formed by São Miguel and Santa Maria Islands. The Azorean climate is characterised by stable temperatures, a high annual rainfall and relative air humidity and persistent winds (e.g. http://www.climaat.angra.uac.pt/). The Azorean landscape has suffered severe modifications as a consequence of human settlement, mainly associated with land-use changes by the replacement of native forests by agricultural fields, forestry plantations, namely of Japanese cedar *Cryptomeriajaponica* D.Don and Australian cheesewood *Pittosporumundulatum* Vent. and urban areas (Gaspar et al. 2008, Triantis et al. 2010). Currently, less than 3% of the Archipelago is covered by pristine forest (Gaspar et al. 2008, Norder et al. 2020), which is restricted to the highest elevations and most inaccessible areas.

The Azores Archipelago harbour regular breeding species such as the Monteiro's storm-petrel, the band-rumped storm-petrel, the Bulwer's petrel, the Cory’s shearwater, the Barolo's shearwater, the Manx shearwater, the yellow-legged gull, the sooty tern, the roseate tern and the common tern. The colonies of these species are generally located on small islets close to the main islands and coastal cliffs dominated by low forests of native and exotic vegetation and rocky/uncovered areas (Del Nevo et al. 1993, Monteiro et al. 1996a, Monteiro et al. 1996b, Monteiro et al. 1999, Bolton et al. 2008).

### Site description

**Figure 1.**
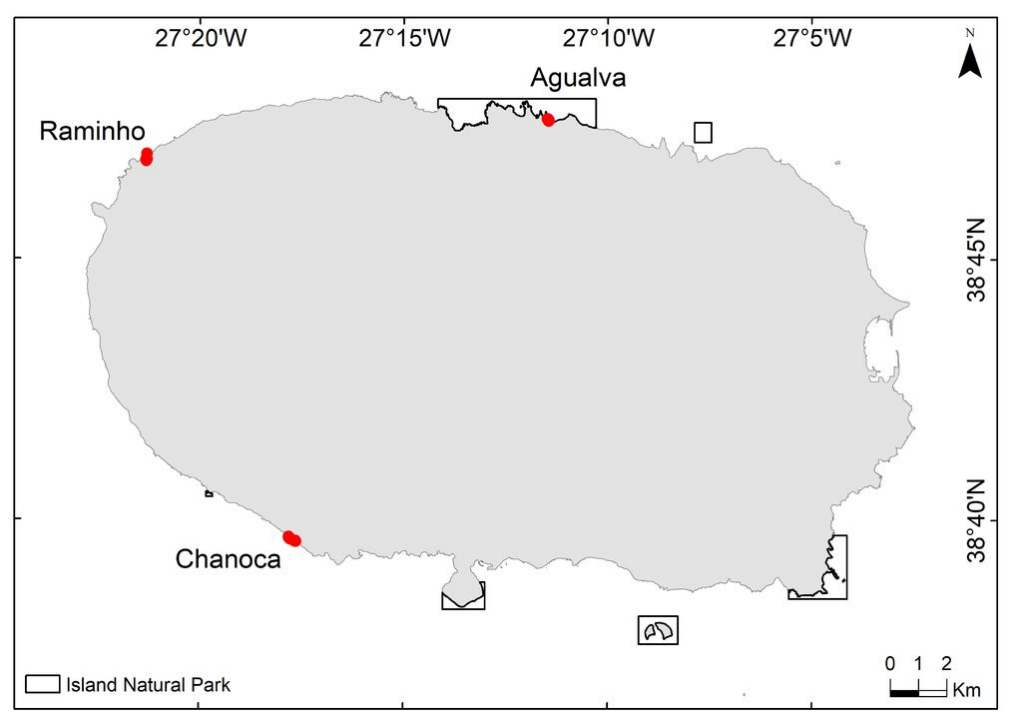
Cory’s shearwater *Calonectrisdiomedea* breeding sites (Agualva, Chanoca and Raminho) monitored between April and October 2019 on Terceira Island (27º10' W­, 38º40' N), Azores, Portugal.

The study was performed on three colonies of Cory’s shearwater, on Terceira Island (27º10' W­, 38º40' N) (Fig. 1).

Chanoca is located in the southern coast of Terceira Island. This area is formed by cliffs enclosing small rocky bays and it is covered by rocky and barren areas and a low density of herbaceous plants. Sour fig *Carpobrotusedulis* (L.) N.E.Br. is the dominant plant in the area, being relatively abundant and widespread. Chanoca is located next to an urban area (< 200 m) and it is frequently used by local fishermen to gain access to the sea.

Raminho is located in the north-western part of Terceira Island. The area is formed by cliffs, dominated by a low forest of native vegetation, namely *Ericaazorica* and small and scarce individuals of *Morellafaya* (Aiton) Wilbur. The nests are located in the rocky cliffs and within the forest. Although the colony is relatively inaccessible, there is a narrow dirt road that goes through the forest to the lowest part of the colony, near to the coast. Raminho is located approximately 700-800 m from the nearest urban area.

Agualva is located in the northern part of Terceira Island. The area consists of a rocky area scarcely covered by native vegetation, mainly *Ericaazorica* Hochst. ex Seub. Although it is relatively isolated (> 1400 m from the nearest urban area), it is crossed by a walking trail (PR2TER - Baías da Agualva).

### Camera-trapping

Fieldwork was performed between 4 April and 25 October 2019, covering the entire breeding season of Cory’s shearwater in the Azores (Monteiro et al. 1996a). At the beginning of the breeding season, we searched for nests in the three aforementioned breeding sites. Once an occupied nest was located, we installed one motion-triggered infrared camera Bushnell Trophy HD, Moultrie 880i and 990i (henceforth referred as cameras). The cameras were fixed on a wooden pole, adjacent shrub or rock and deployed at 50-100 cm from the nest entrance. Cameras were configured to take 8 MB-photos, with 30 s of delay between records (Lamelas-López et al. 2020a). Date and time were automatically recorded for each photo. Cameras recorded continuously during the entire breeding season. If a nest was predated, the camera was moved to another occupied nest. A total of 37 nests were monitored using camera-traps (14 in Chanoca, 13 in Raminho and 10 in Agualva), but two cameras in Chanoca were stolen and it was impossible to retrieve any information from those nests. Nests were visually inspected every 10 days to verify nest predation, camera functioning and to replace cameras SD cards and batteries when necessary.

From camera-trap records, we obtained the Relative Abundance Index (RAI) (O'Connell et al. 2011) of both Cory’s shearwater and its potential predators. The RAI was calculated as the number of records of a species during a sampling effort of 100 camera-trap days (Lamelas-López et al. 2020a, Lamelas-López and Marco 2020).

### Nest characteristics and habitat variables

For each nest, we measured the maximum width and height of the cavity entrance, the nest entrance area (calculated by multiplying nest width and height) and the nest depth (distance from the entrance to the back of the cavity) (see Hervías et al. (2013b)). We also visually estimated the amount of nest entrance covered by vegetation, as a proxy of nest visibility and accessibility.

In addition, we calculated the cumulative proportion of covered (trees, shrubs and herbs cover) and uncovered areas (cover of rocky and barren areas) within a radius of 25 m from the nest. Nest UTM coordinates were recorded using a GPS navigator (Garmin eTrex). We also measured the distance from the nest to the nearest urban area using the ArcGIS software (ESRI 2011).

### Statistical analysis

We calculated the daily mortality rate (DME) as (*P/D*), where *P* stands for “Predation” and is the status of the nest at the end of the breeding season (i.e. predated or not predated) and *D* stands for “Days” and is the number of days when the camera was recording. Consequently, the daily mortality rate is a continuous value between 0 (i.e. the nest survived) and 1 (i.e. the nest was predated after the first day of recording). The DME was used as a response variable in two zero-inflated beta-regressions; one including only abiotic factors and the other only biotic factors. The beta-regression distribution is suitable for continuous proportional data with a high frequency of zeroes, as in our dataset. Log-transformation was applied to all variables with outliers (i.e. nest area; nest depth; distance from the nearest urban centre and the RAIs of *Rattus* sp.). Before creating the models, we calculated the Pearson Correlation Coefficient (r) to test for potential correlation between the abiotic factors and, independently, between the biotic factors.

Since we found high correlation between nest area and nest depth (r = 0.45, Suppl. material 1) and between the distance from the nearest urban centre and percentage of vegetation near the nest (r = 0.44, Figures S1), the model including abiotic factors included only the log-transformed distance from the nearest urban centre and the log-transformed nest area as fixed factors and the site included as a random factor. No issue was detected in the model residuals (Suppl. material 2).

No concerning correlations were detected between biotic factors (Suppl. material 3). Nevertheless, since most nests were presumably predated by rats, we only included the log-transformed RAI of *Rattus* sp. as a fixed factor and the site as a random factor. No issue was detected in the model residuals (Suppl. material 4).

Moreover, we tested which factors affected the activity of rats around Cory’s shearwater nests formulating a linear model were the log-transformed RAI of *Rattus* sp. was the response and the presence of vegetation at the entrance of the nest (binary), the percentage of vegetation near the nest, the log-transformed nest area, the log-transformed nest depth, the log-transformed distance from the nearest urban centre and the RAIs of potential predators of rats (cat and least weasel) were the fixed factors and the site was the random factor. This model was simplified using stepwise selection with the R function *step*. The final model included the presence of vegetation at the entrance of the nest, the percentage of vegetation near the nest and the log-transformed distance from the nearest urban centre and did not show any concerning pattern in the residuals (Suppl. material 5).

An overview of the model can be found in Table 1. Models were created using the R packages glmmTMB (Brooks et al. 2017), validated using DHARMa (Hartig 2022) and visualised using ggeffects (Lüdecke 2018). All analyses were performed in R (RCoreTeam 2023).

## Results

**Figure 2.**
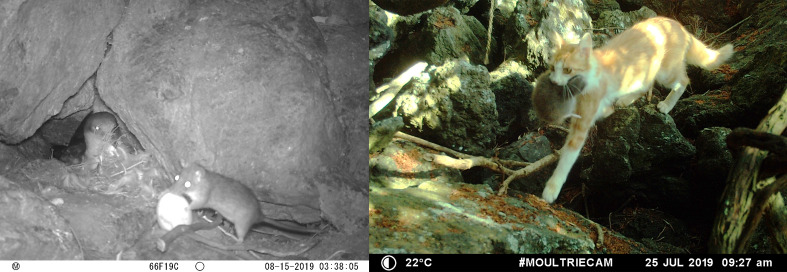
Photos of predation events on Cory’s shearwater nests. Left: black rat preying on an egg in Raminho; Right: cat preying on a chick in Agualva.

A total of 292,624 photos were obtained, of which 286,061 (97.7%) portrayed Cory’s shearwaters, 5045 (1.7%) non-target species (e.g. other birds, rabbits) and 1518 (0.52%) potential predators (Table 1). Overall, nine of the thirty-five successful monitored nests (25.7%) across the three monitored colonies were predated throughout the breeding season. Of these, two were predated by black rats (22.2%) and one (11.1%) by feral cats. Predators were not identified in six nests (66.7%), but the high activity of rats in these nests suggests that they were the most likely predators (Table 2; Fig. 2).

The highest nest predation was recorded in Agualva (33.3% of the nests), followed by Raminho (30.8%) and Chanoca (15.4%). The mean RAI of Cory’s shearwater in nests that were predated (12,798.2) was higher than in nests that were not predated (9806.0). The same was true for the RAI of recorded predators, such as black rats (81.8 vs. 29.1 for predated and not predated nests, respectively) and cats (8.4 vs. 6.9), but not for potential predators of eggs or chicks such as the least weasel (0.0 vs. 0.3) and the house mouse (8.1 vs. 10.8).

**Figure 3.**
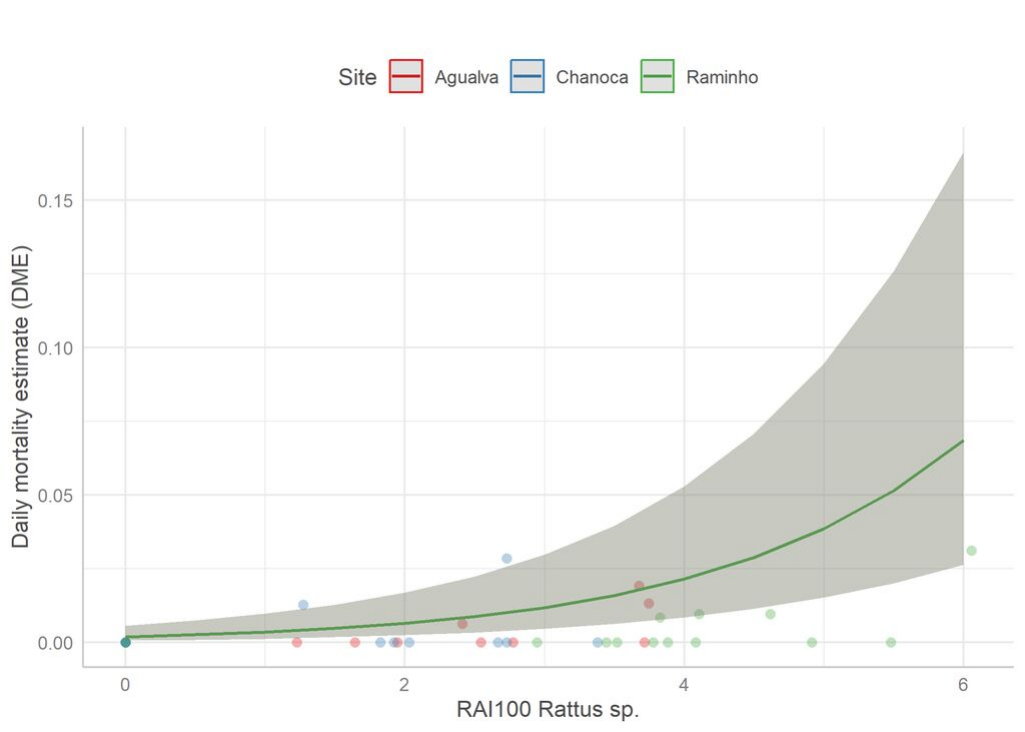
Relationship between daily mortality estimate (DME) of Cory`s shearwater nests and log-transformed Relative Abundance Index of black rats (RAI100 *Rattusrattus*).

No abiotic factor significantly affected the DME, while the log-transformed RAI of *Rattus* sp. was significantly and negatively related to the nest survival (p < 0.001, GLMM; Fig. 3; Table 3).

**Figure 4.**
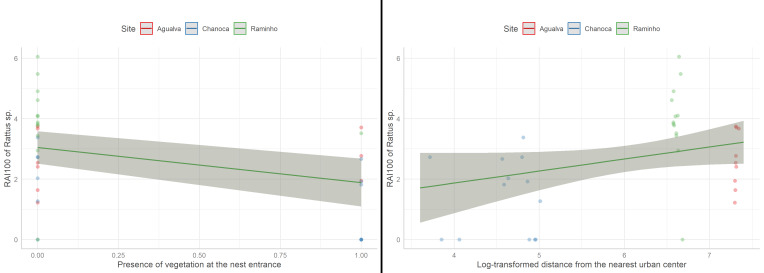
Relationship between log-transformed Relative Abundance Index of *Rattus* sp. and the presence of vegetation at the nest entrance (Left) and the log-transformed distance from the nearest urban centre (Right).

The best model explaining the RAI of *Rattus* sp. included the percentage of vegetation nearby the nest (despite this factor being non-significant), the log-transformed distance from the nests to the urban areas and the presence of vegetation at the entrance of the nest. The RAI of *Rattus* sp. was marginally significantly (p = 0.07, LM; Fig. 4 - Right) and positively related to the log-transformed distance from the nearest urban centre and significantly (p = 0.02, LM; Fig. 4 - Left) and negatively related to the presence of vegetation at the entrance of the nest.

## Discussion

The impacts of rodents, particularly rats and cats on insular biota have been widely documented (Courchamp et al. 2003, Towns et al. 2006, Jones et al. 2008, Oppel et al. 2010, Medina et al. 2013, Doherty et al. 2016, Russell et al. 2017). These species have been reported as major seabird and terrestrial bird predators in many oceanic islands worldwide (e.g. Long (2003), Jones et al. (2008), Dumont et al. (2010), Russell et al. (2017)). Our results show that black rats and cats, both included in the top 100 invasive species list (Lowe et al. 2000), are the main predators of Cory’s shearwater juveniles in the Azores. Specifically, we observed instances of black rats preying on eggs, as well as a single cat capturing a young chick of Cory's shearwater. Our findings are in accordance with the results of Hervías et al. (2013b) for Corvo Island, Azores. However, Hervías et al. (2013b) found that the cat was the main predator, while on Terceira our results support a predation dominance by the black rat. Rodents have been identified as major predators of *Calonectris* shearwaters in other parts of the world. For instance, black rats were the main cause of breeding failure (up to 85% in some years) in Corsica (*Calonectrisdiomedea*; Thibault (1995)) and Norway rats were the main cause of breeding failure (83.9% of breeding failure) in Sasudo Island, Japan (*Calonectrisleucomelas*; Lee and Yoo (2002)). Feral cats and black rats are also the main predators of other seabirds (Del Nevo et al. 1993, Monteiro et al. 1996a, Amaral et al. 2010, Lamelas-López et al. 2021) and terrestrial bird species (Alcover et al. 2015, Lamelas-López et al. 2020a) in the Azores, Canary Islands (Hernández et al. 1990, Hernández et al. 2008, Medina and Nogales 2008), Madeira (Menezes and Oliveira 2002) and Cabo Verde (Semedo et al. 2020, Medina et al. 2020).

The abundance of introduced mammal predators (Thibault 1995, Igual et al. 2006), as well as nest characteristics, are known to affect Cory’s shearwater nest success (Granadeiro 1991, Thibault 1995, Igual et al. 2006, Bourgeois and Vidal 2007). We found that the relative abundance of black rats in Cory’s shearwater nests was the main factor explaining nest mortality. This variable was significantly and negatively related with the daily survival estimate of Cory’s shearwater nests. This observation contrasts with the results of Hervías et al. (2013b), who found that nest survival increased with black rat activity, suggesting that rats are an alternative food resource to the main predator, the cat. This supports the mesopredator release effect hypothesis, which states that a secondary predator can increase its abundance when the top-predator is removed (Courchamp et al. 2003, Russell et al. 2009). On the other hand, our results are consistent with Lamelas-López and Santos (2021), who found that bottom-up regulation is the major driver of predator abundance (rodents, cats and mustelids) in the Azores. Contrary to Corvo Island, where cats are the only top-predator, several large predators are present on Terceira Island, which might explain why our study does not support the results of Hervías et al. 2013b. The other variable affecting the relative abundance of black rats was the vegetation cover at the nest entrance. This variable was not significantly related with the survival, but was significantly and negatively related to the relative abundance of black rats, which may be explained by the fact that the vegetation at the nest entrance protects the nest, making it less accessible and detectable. The distance to the urban areas was positively and marginally significantly related with the relative abundance of black rats. This is in accordance with the idea that the introduction and establishment success of invasive species are directly linked to human activities (Russell et al. 2017).

Other potential nest predators of Cory’s shearwater eggs and chicks include house mice and mustelids. In our study, we detected both species in the nests, but predation was never recorded. The impact of the house mouse as bird nest predator has rarely been reported for the Azores (Hervías et al. 2013) and Madeira (Zino et al. 2008), while that of mustelids is yet to be assessed, although they are often reported as potential threats to both seabirds (Del Nevo et al. 1993, Neves et al. 2011, Lamelas-López et al. 2020b) and terrestrial birds (Fontaine et al. 2019).

## Conclusions

The identification of the main nest predators is crucial for the management and conservation of native bird populations (Sanders and Maloney 2002, Rader et al. 2007, Richardson et al. 2009). This is particularly relevant for oceanic islands, which harbour an important number of threatened and endemic species (Whittaker and Fernández-Palacios 2007). Our study also indicates that the main predators can vary amongst Azores islands, possibly depending on the complexity of the local trophic network. These results highlight the importance of conducting thorough preliminary studies before any eradication attempts to evaluate trade-offs. Our results showed that the removal of feral cats from Terceira would benefit bird conservation and would not impact black rats, as the relative abundance of cats does not seem to be related with that of black rats (Lamelas-López and Santos 2021). The coordinated removal of feral cats and the control of black rats could potentially maximise conservation gains for Cory’s shearwater, in particular and for Azorean native birds, in general.

## Supplementary Material

CECFCCA1-D54E-531F-9F96-3F6CC9B5A5E5 10.3897/BDJ.11.e112871.suppl1 Supplementary material 1 Pearson Correlation Coefficient between the abiotic factors Data type Figure Brief description Pearson Correlation Coefficient between the abiotic factors: the log-transformed distance from the nearest urban centre (logDistUrb), the log-transformed nest area (logArea), the log-transformed nest depth (logDepth), the percentage of vegetation near the nest (Covered) and the presence of vegetation at the nest entrance as a binary variable (Veg_Entr2). File: oo_930342.tiff https://binary.pensoft.net/file/930342 Lucas Lamelas-Lopez, Marco Ferrante, Paulo A.V. Borges, Isabel A. Rosario, Veronica Neves

0D2DE743-1A36-59EB-B4E0-827DB404E322 10.3897/BDJ.11.e112871.suppl2 Supplementary material 2 Model validation plot of the daily mortality rate model including abiotic factors Data type Figure Brief description Model validation plot created using the simulateResiduals function in DHARMa of the daily mortality rate model including abiotic factors. File: oo_930345.tiff https://binary.pensoft.net/file/930345 Lucas Lamelas-Lopez, Marco Ferrante, Paulo A.V. Borges, Isabel A. Rosario, Veronica Neves

609EC321-E6D6-5346-AAAD-C8CE218D82CA 10.3897/BDJ.11.e112871.suppl3 Supplementary material 3 Pearson Correlation Coefficient between the biotic factors Data type Figure Brief description Pearson Correlation Coefficient between the biotic factors: the Relative Abundance Index (RAI) of the Cory’s shearwater (RAI_Cd), the log-transformed RAI of *Rattus* sp. (logRAIrr), the log-transformed RAI of the least weasel (logRAImn), the RAI of the domestic cat (RAI_Fc) and the RAI of the house mouse (RAI_Mm). File: oo_930346.tiff https://binary.pensoft.net/file/930346 Lucas Lamelas-Lopez, Marco Ferrante, Paulo A.V. Borges, Isabel A. Rosario, Veronica Neves

21CB6836-E9FF-5207-9E81-DAB6D3652875 10.3897/BDJ.11.e112871.suppl4 Supplementary material 4 Model validation plot of the daily mortality rate model including biotic factors Data type Figure Brief description Model validation plot created using the simulateResiduals function in DHARMa of the daily mortality rate model including biotic factors (i.e. the log-transformed RAI of *Rattus* sp.). File: oo_930347.tiff https://binary.pensoft.net/file/930347 Lucas Lamelas-Lopez, Marco Ferrante, Paulo A.V. Borges, Isabel A. Rosario, Veronica NevesLucas Lamelas-Lopez, Marco Ferrante, Paulo A.V. Borges, Isabel A. Rosario, Veronica Neves

A705979D-0516-5C8F-B7B2-BA01C501156C 10.3897/BDJ.11.e112871.suppl5 Supplementary material 5 Model validation plot of the log-transformed RAI of *Rattus* sp. model Data type Figure Brief description Model validation plot created using the simulateResiduals function in DHARMa of the log-transformed RAI of *Rattus* sp. model including the presence of vegetation at the entrance of the nest, the percentage of vegetation near the nest and the log-transformed distance from the nearest urban centre. File: oo_930348.tiff https://binary.pensoft.net/file/930348 Lucas Lamelas-Lopez, Marco Ferrante, Paulo A.V. Borges, Isabel A. Rosario, Veronica Neves

## Figures and Tables

**Table 1.** Overview of the statistical models with the daily mortality estimates (DME) and the log-transformed RAI of *Rattus* sp. (logRAIrr) as responses. Fixed factors are coded as follows: log-transformed nest area (logArea), log-transformed distance from the nearest urban centre (logDistUrb), log-transformed RAI of *Rattus* sp. (logRAIrr), presence of vegetation at the entrance of the nest (VegEntr), the percentage of vegetation near the nest (Cover).

**Model**	**Response**	**Distribution**	**Fixed effects**	**Random effects**
Abiotic factor	DME	Zero-inflated beta distribution	logArea + logDistUrb	Site
Biotic factor	DME	Zero-inflated beta distribution	logRAIrr	Site
RAI *Rattus* sp.	logRAIrr	Gaussian	VegEntr + Cover + logDistUrb	Site

**Table 2.** Detected potential predator species, prey age-class, total number of photos recorded, number of predated nests and mean Relative Abundance Index (RAI) of potential predators. *Black rats were only recorded predating two nests, but the high RAI detected nearby the other predated nests suggest that they were likely responsible for the predation of eight nests; N/A – Non applicable.

Predator	Prey age-class	Number of records	Number of predated nests	Mean RAI
Domestic cat - *Feliscatus*	Chick	176	1	7.26
Black rat - *Rattusrattus*	Egg	973	8*	42.62
House mouse - *Musmusculus*	N/A	363	0	10.10
Least weasel - *Mustelanivalis*	N/A	6	0	0.20

**Table 3.** Outputs of the three statistical models with daily mortality estimates (DME) or the log-transformed RAI of *Rattus* sp. (logRAIrr) as response variables.

**Response**	**Conditional model**	**Variance**	**SD**		
DME	*Site*	6.42E-11	8.01E-06		
		**Estimate**	**SE**	**z-value**	**Significance**
	*Intercept*	-3.56791	1.80982	-1.971	0.0487 *
	*logDistUrb*	-0.18838	0.16936	-1.112	0.266
	*logArea*	0.08966	0.17658	0.508	0.6116
	**Zero-inflation model**				
	*Intercept*	1.0609	0.3867	2.743	0.00609 **
DME	*Site*	6.70E-01	8.19E-01		
		**Estimate**	**SE**	**z-value**	**Significance**
	*Intercept*	-6.2332	0.55015	-11.33	< 2e-16 ***
	*logRAIrr*	0.60395	0.07612	7.934	2.12e-15 ***
	**Zero-inflation model**				
	*Intercept*	1.0609	0.3867	2.743	0.00609 **
logRAIrr	*Site*	4.29E-09	6.55E-05		
		**Estimate**	**SE**	**z-value**	**Significance**
	*Intercept*	-0.35059	1.2469	-0.281	0.779
	*Cover*	0.01662	0.01149	1.446	0.148
	*VegEntr*	-1.15858	0.49786	-2.327	0.020 *
	*logDistUrb*	0.39758	0.21946	1.812	0.070 .
