# Supplementary figures and images for: Nest predation of Cory's shearwater Calonectrisborealis (Aves, Procellariiformes) by introduced mammals on Terceira Island, Azores
Source: Biodivers Data J. 2023 Dec 22;11:e112871. doi: 10.3897/BDJ.11.e112871 (PMC10757304; doi:10.3897/BDJ.11.e112871)

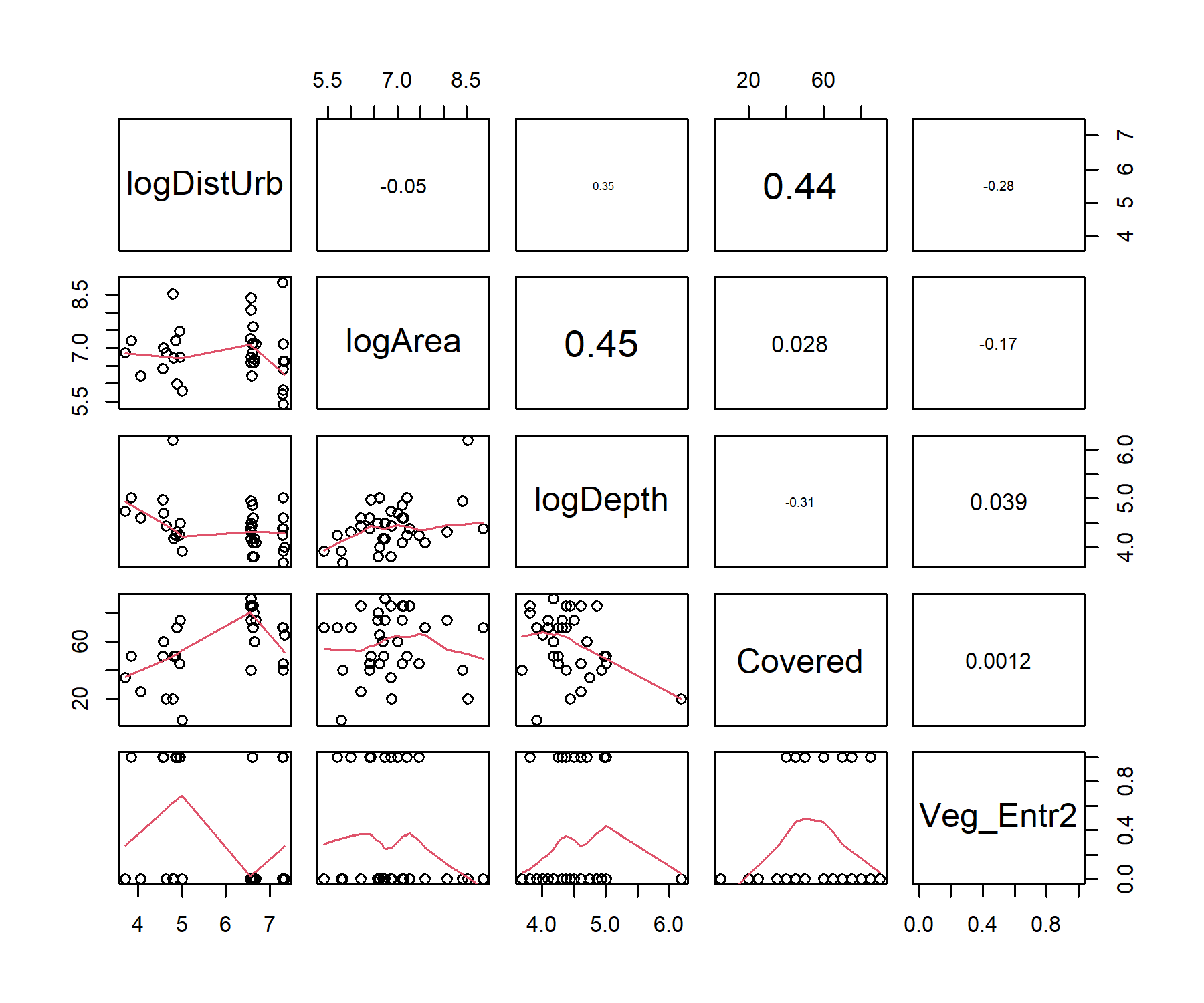

Supplement: Supplementary material 1 — Pearson Correlation Coefficient between the abiotic factors [file bdj-11-e112871-s001.tiff]

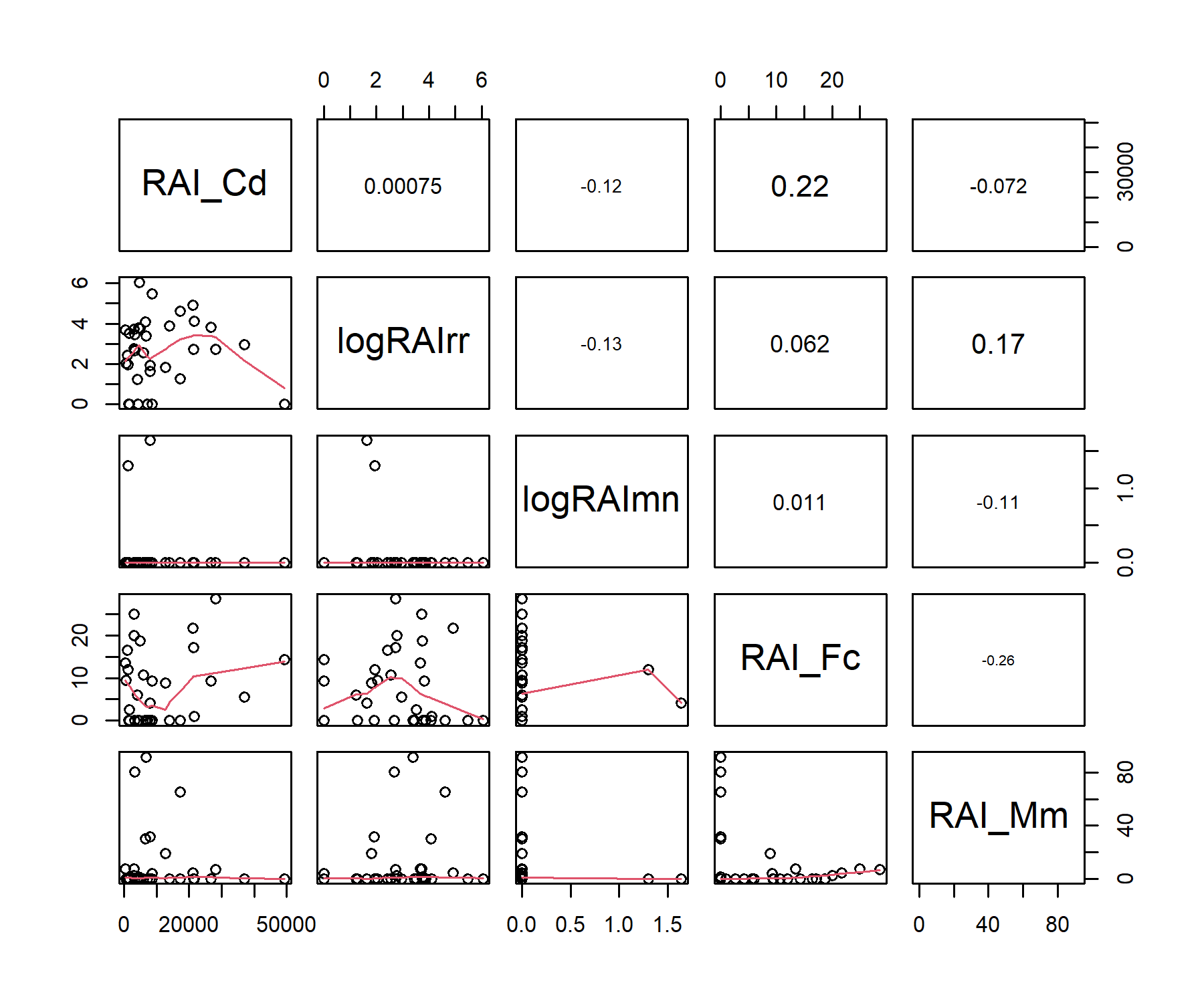

Supplement: Supplementary material 2 — Model validation plot of the daily mortality rate model including abiotic factors [file bdj-11-e112871-s002.tiff]

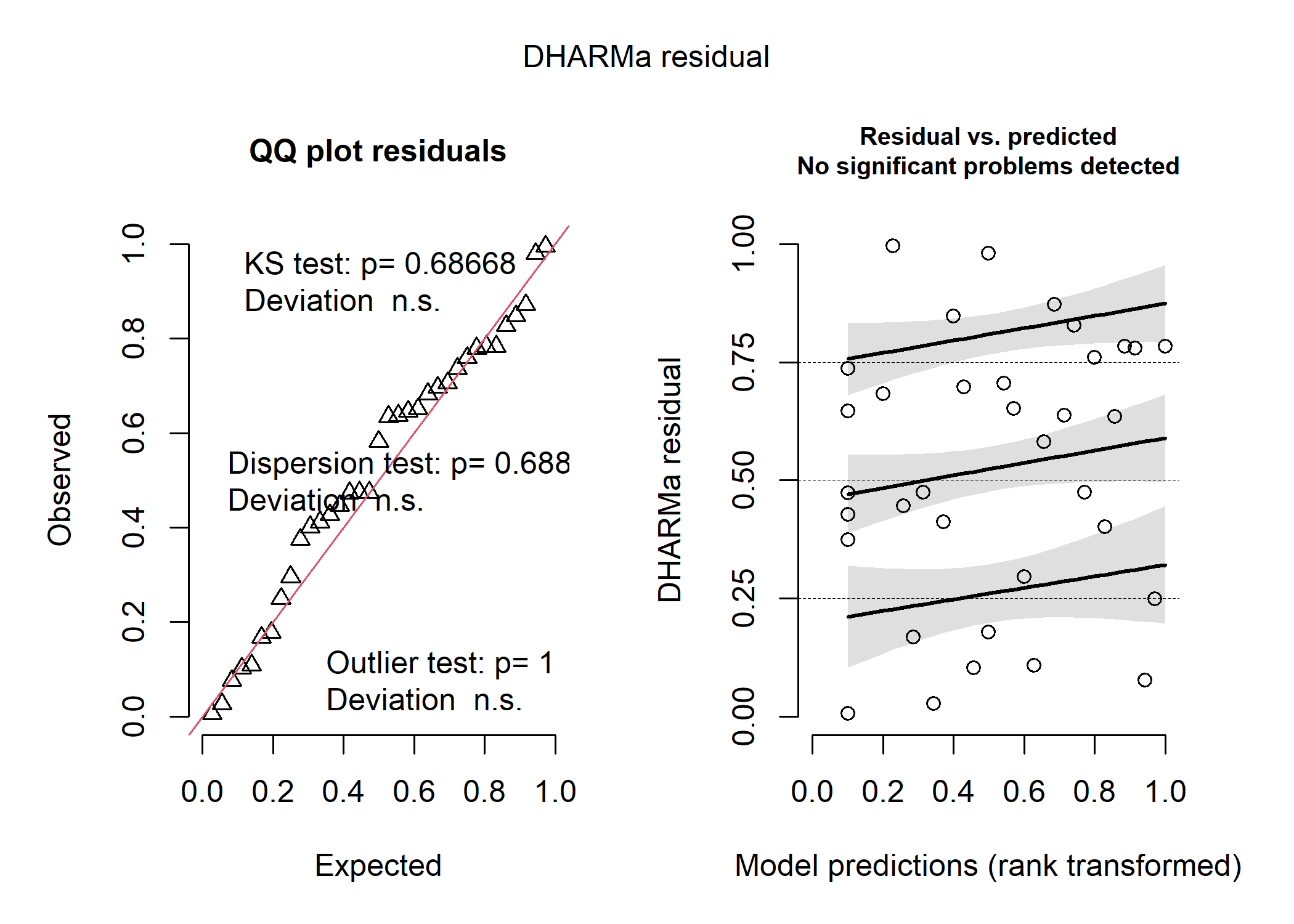

Supplement: Supplementary material 4 — Model validation plot of the daily mortality rate model including biotic factors [file bdj-11-e112871-s004.tiff]

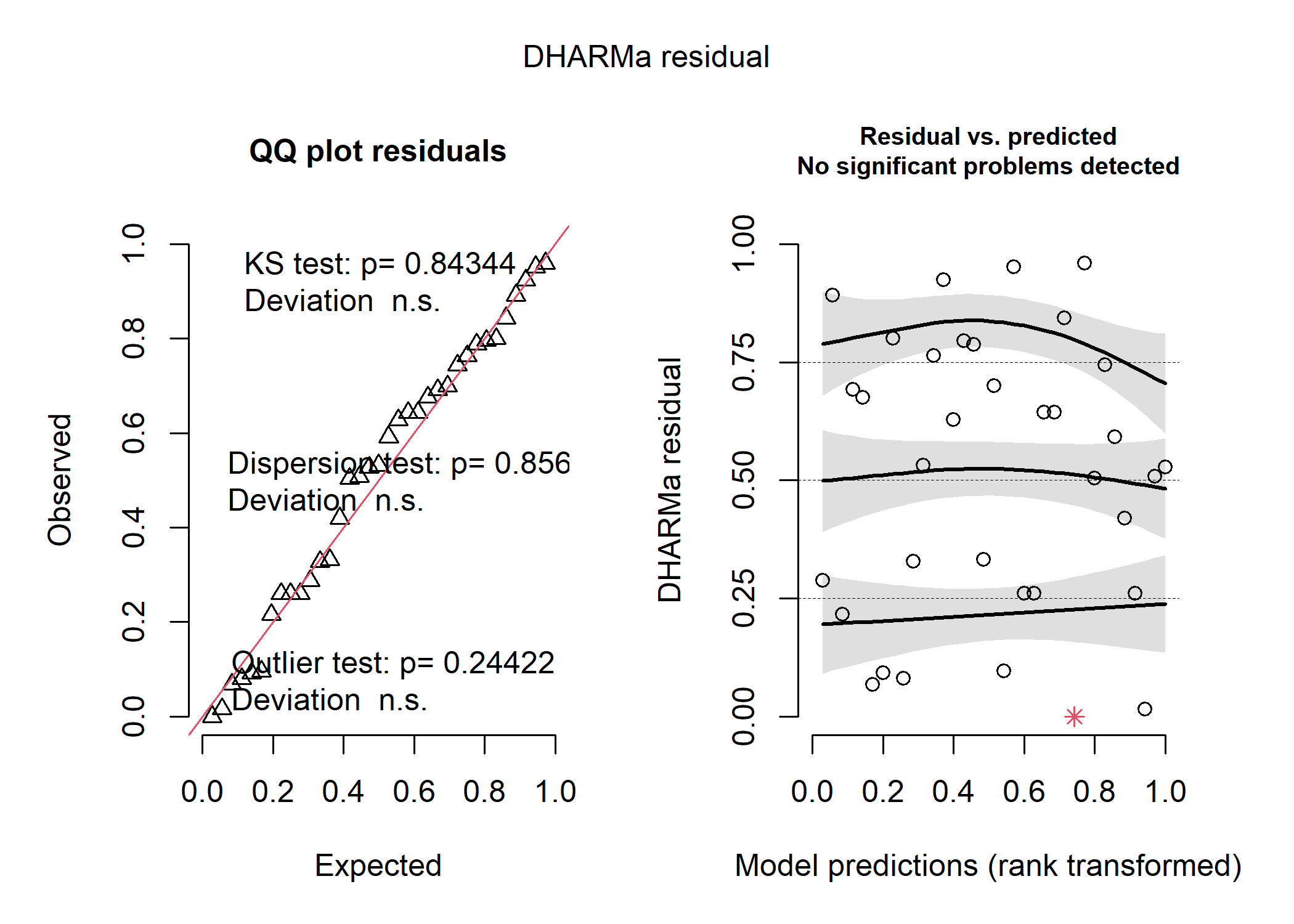

Supplement: Supplementary material 5 — Model validation plot of the log-transformed RAI of Rattus sp. model [file bdj-11-e112871-s005.tiff]
